# Automatic classification of mobile apps to ensure safe usage for adolescents

**DOI:** 10.1371/journal.pone.0313953

**Published:** 2025-01-16

**Authors:** Hanadi Hakami

**Affiliations:** Department of Software Engineering, Jeddah College of Engineering, University of Business and Technology, Jeddah, Saudi Arabia; Zayed University, UNITED ARAB EMIRATES

## Abstract

The integration of mobile devices into adolescents’ daily lives is significant, making it imperative to prioritize their safety and security. With the imminent arrival of fast internet (6G), offering increased bandwidth and reduced latency compared to its predecessor (5G), real-time streaming of high-quality video and audio to mobile devices will become feasible. To effectively leverage the fast internet, accurately classifying Mobile Applications (M-APPs) is crucial to shield adolescents from inappropriate content, including violent videos, pornography, hate speech, and cyberbullying. This work introduces an innovative approach utilizing Deep Learning techniques, specifically Attentional Convolutional Neural Networks (A-CNNs), for classifying M-APPs. The goal is to secure adolescent mobile usage by predicting the potential negative impact of M-APPs on adolescents. The proposed methodology employs multiple Machine and Deep Learning (M/DL) models, but A-CNNs based on Bidirectional Encoder Representations from Transformers embeddings outperformed other models, achieving an average accuracy of 88.74% and improving the recall from 99.33% to 99.65%.

## 1 Introduction

The utilization of Mobile Applications (M-APPs) by adolescents has witnessed a notable increase across various digital devices, with smartphones experiencing a surge in popularity in recent years [1–4]. However, the comprehensive understanding of the effects of M-APP usage on adolescents remains incomplete and can vary depending on the types and patterns of usage. Some research indicates that educational apps can have a positive impact on the learning and development of adolescents [5]. These M-APPs contribute to fostering new skills and concepts engagingly and interactively. Conversely, the use of inappropriate M-APPs has been associated with adverse effects on adolescents [6]. Additionally, excessive use of M-APPs may result in reduced physical, social, and mental well-being among adolescents [7–9]. Another significant concern is that certain M-APPs have the potential to expose adolescents to inappropriate content, including violence and adult themes [10].

Using parental control apps, people can monitor and restrict their children’s activities to the internet and not suitable M-APPs on their mobile devices or computers [11]. By using parental control apps, parents can protect their adolescents from potential dangers that may be present on the internet, i.e., violence, pornography, hate speech, cyberbullying, and online predators. Even though it has been shown that these apps are rarely utilized, and their adoption rate is very low [12, 13]. Many discrepancies exist in M-APPs’ age suitability ratings because of the lack of standardization in M-APPs’ rating process [14–16]. Moreover, different countries and regions may have different regulations and guidelines for determining the age suitability of M-APPs. Additionally, M-APP usage can change over time, making it difficult to assign a fixed age rating that is always consistent. Consequently, these elements lead to inconsistent age ratings across different M-APP stores and regions [17].

With 6G, bandwidth and latency will be greatly improved over its predecessor, 5G. Consequently, real-time streaming of top-notch video and audio content to mobile devices will be achievable. However, to harness the full potential of 6G effectively, it becomes crucial to categorize mobile apps precisely. This will ensure effective protection for adolescents against inappropriate content. It is important to design techniques that can automatically predict M-APPs having a negative impact on adolescents. Predicting M-APPs that harm adolescents is by taking into factors, e.g., account user behavior, in-app context, and M-APP rating. Moreover, M-APPs’ user reviews can indicate unsuitable content for adolescents, i.e., the presence of violence, sexual content, or offensive language [18–20].

For the abovementioned purpose, an innovative approach utilizing Deep Learning (DL) techniques, specifically Attentional Convolutional Neural Networks (A-CNN), is presented for classifying M-APPs aimed at securing adolescent mobile usage to predict the potential negative impact of M-APPs on adolescents. Text classification tasks commonly use Convolutional Neural Network (CNN) algorithms [21]. CNN model can learn patterns in the descriptions and categorize them into predefined categories or labels [22]. In the proposed approach, Natural Language Processing (NLP) strategies are used to preprocess information from M-APP user reviews. Next, BERT (Bidirectional Encoder Representations from Transformers) embeddings are calculated and the A-CNN model is trained and tested leveraging these embeddings. The suggested model attained an average accuracy of 88.74%, precision of 88.91%, recall of 99.65%, and F1 score of 93.96%. The significant contributions of this paper are as follows:

- Introducing an innovative attentional convolutional neural network designed for the automatic classification of M-APPs to enhance adolescent safety. This automation will aid parents and M-APP stores in identifying and blocking harmful M-APPs, thereby protecting adolescents from inappropriate content. Moreover, M-APP stores can implement A-CNN to improve the M-APP’s rating process and to avoid inconsistent age ratings.
- Introducing an automated algorithm that calculates BERT embeddings without requiring feature engineering.
- Perform and evaluate the effectiveness of the suggested model toward M-APP classification on the dataset collected from *HuggingFace* (dataset available: https://huggingface.co/datasets/app_reviews/blob/main/app_reviews.py) and compared to other ML models.

The paper is structured as follows: Section 2 provides a general summary of the research background. In Section 3, the details of the proposed approach are presented. The evaluation methods and results are discussed in Section 4. Lastly, the article is concluded in Section 5.

## 2 Related work

Machine/Deep Learning (M/DL) has been broadly leveraged in many NLP tasks, and it has shown to be effective in text classification, language translation, language generation, and sentiment analysis [23, 24]. However, DL models have been shown to achieve state-of-the-art performance in many NLP tasks [25–27]. Many M/DL algorithms are implemented to monitor and track the interactions of children and adolescents with M-APPs [15, 28, 29] as discussed in this concise summary but very little literature is available concerning mobile app classification for securing adolescents.

Hu et al. [30] proposed a multi-label classification method to identify and categorize mature app content using deep learning and support vector machine. They extracted features and captured label correlations from app descriptions. They evaluated the proposed approach and compared it to other methods using data gathered from the App Store and Google Play Store. The proposed approach achieved 85% precision in identifying mature content and 79% precision in categorizing maturity levels, using only app descriptions.

Meng et al. [31] proposed an ML-based approach to predict the suitability of M-APP for children. They exploited NLP techniques to analyze user reviews of M-APPs, then created a feature vector for each app by performing feature extraction on the bag of words (BOW), i.e., identifying abusive words. The proposed approach was evaluated using a 10-fold cross-validation approach and yielded results with an average precision of 92.76%, recall of 99.33%, and F1-score of 95.93%.

Liu et al. [32] proposed an SVM-based ML model for identifying M-APPs that are designed for children. It was tested on 1,728 apps from Google Play and found to be 95 percent accurate. Nearly 68,000 apps for kids were identified using the model based on a set of nearly 1 million free apps from Google Play. As part of the study, they analyzed the privacy-related behaviors of each app by analyzing the third-party libraries used.

Zhou et al. [33] used a combination of traditional ML algorithms, i.e., naive Bayes, decision tree, random forest, and SVM, along with deep learning algorithms, i.e., CNN and Recurrent Neural Network (RNN) to identify incorrect maturity ratings of M-APPs. In addition to the app market, they also incorporated features from M-APPs. They discovered that their method gave them 96.98% accuracy, 96.21% precision, and 97.80% recall when they tested it in the market testing. For tests cross-market, it still performed decently, with 88.74% accuracy, 98.75% precision, and 83.72% recall.

Serra et al. [34] proposed a self-monitoring control using a CNN to identify the appropriateness of the content. The model achieved a high F1 score for appropriate and inappropriate content of 98.95% and 98.94%, respectively.

In conclusion, various issues related to inappropriate M-APPs for kids have been identified by researchers, and various M/DL solutions have been proposed to address these issues. However, the automatic M-APP classification for securing adolescents remains an important area of focus, especially with the significant advancement in bandwidth introduced by 6G.

### 2.1 Significance of the study

**Fig 1:**
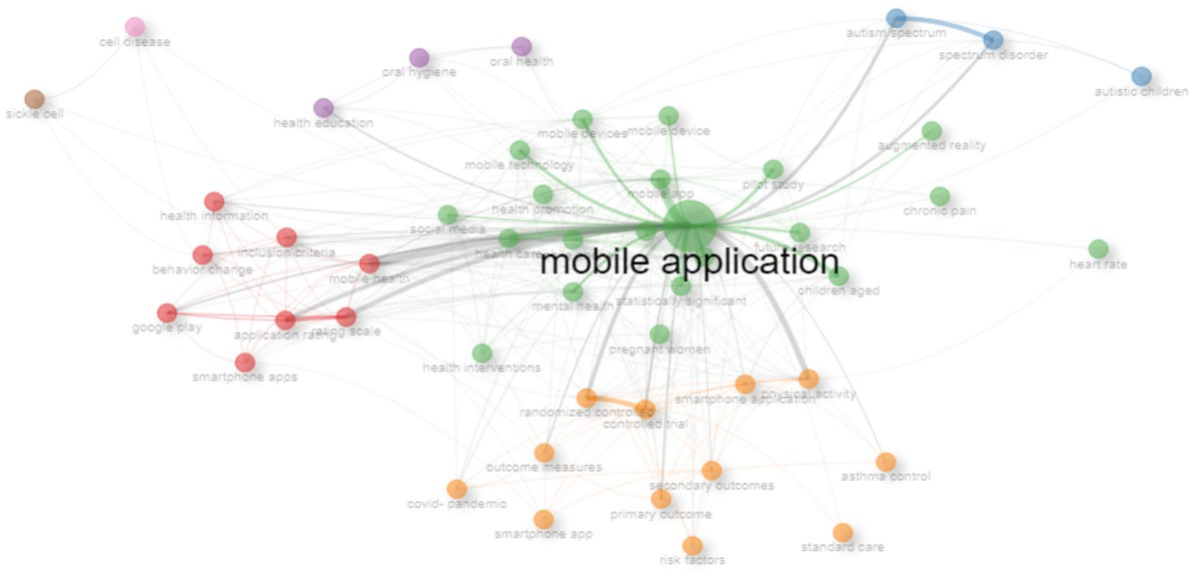
Co-occurrence network.

**Fig 2:**
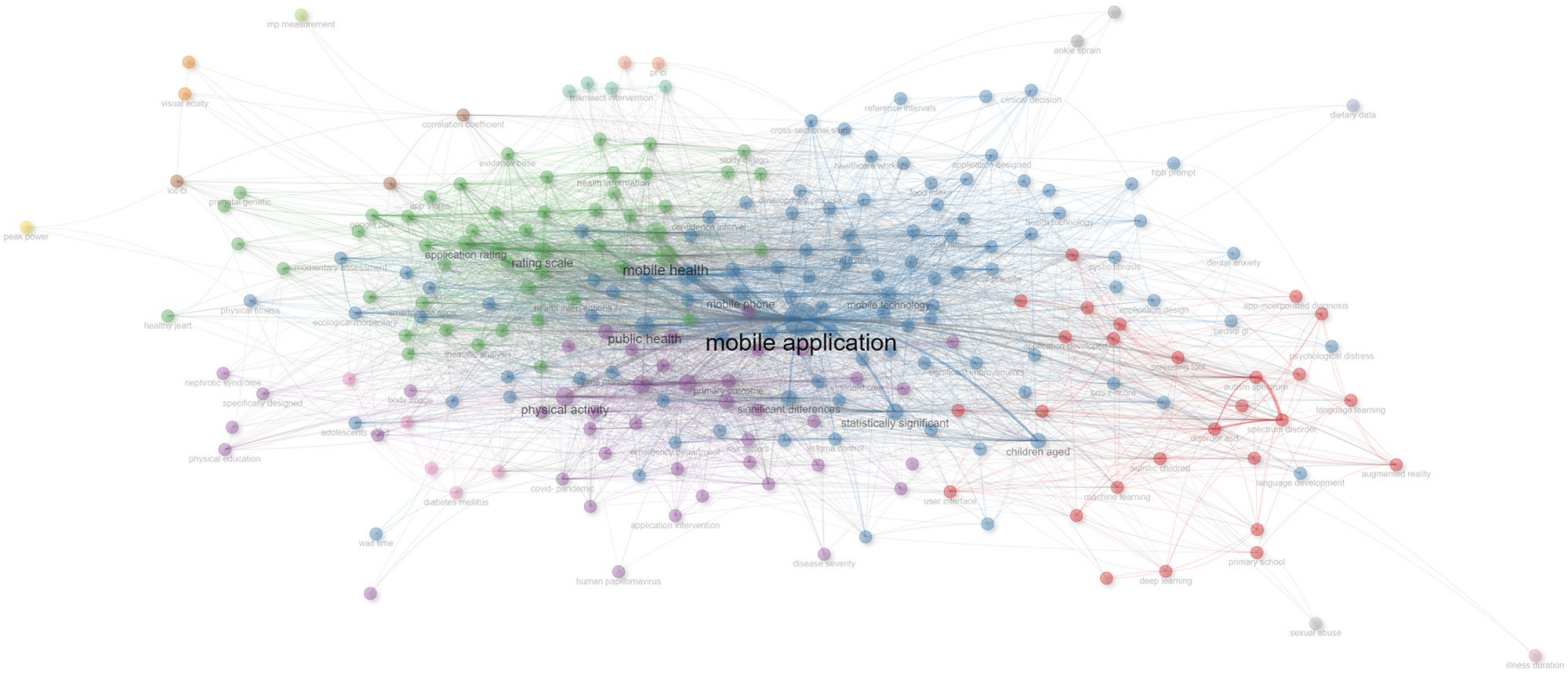
Thematic map.

For a deeper evaluation of the importance of this tpoic, a comprehensive statistical analysis is conducted of the existing literature using the R language. The bibliographic information from 535 highly relevant papers published since 2019 is carefully selected and arranged, sourcing these from the renowned ‘Web of Science’ database. This diverse collection encompasses materials from 99 distinct sources, comprising 484 journal papers, 6 conference proceedings, and 45 review articles, amassing a total of 5,095 citations (excluding self-citations) contributed by 3,194 authors. With this substantial dataset at my disposal, a rigorous statistical analysis is performed, yielding insights into the co-occurrence network and thematic mapping, as depicted in Figs 1 and 2.

Analyzing the data presented in Figs 1 and 2, it becomes evident that the classification of M-Apps has been the subject of extensive scrutiny over several decades. However, notably absent is a focus on identifying appropriate M-Apps to secure adolescence, a critical component of achieving sustainable development goals. In the contemporary landscape, characterized by the ubiquity of mobile platforms, the usage and maintenance of M-Apps have assumed heightened importance. It is imperative to underscore the significance of accurately classifying M-Apps, particularly when considering the unique needs of adolescents. Hence, this research seeks to address this critical aspect by ensuring the precise classification of M-Apps.

## 3 Proposed approach

### 3.1 Overview

**Fig 3:**
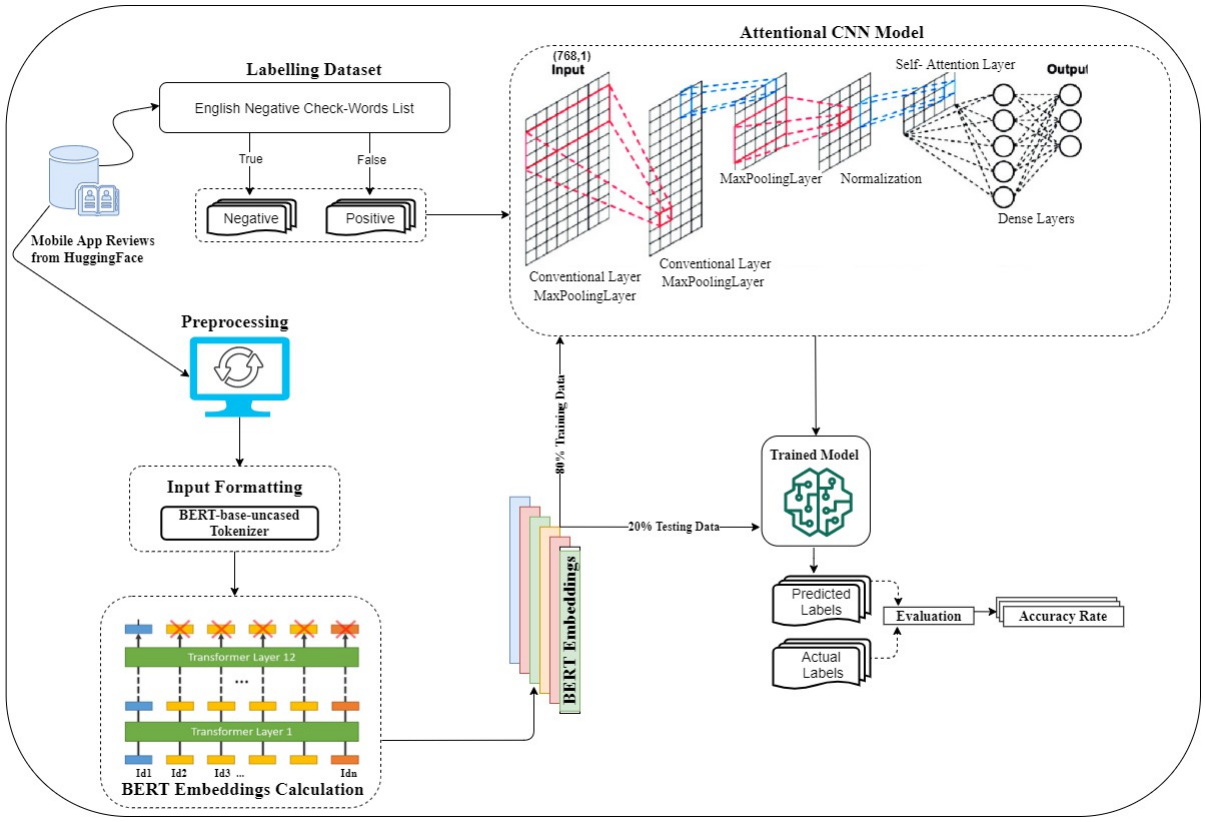
Overview of the proposed approach.

The proposed approach involves the classification of M-APP reviews into two categories, namely *Positive* or *Negative*. The input to the model is an M-APP review, and its output accurately predicts whether the given M-APP is positive for kids or not. Fig 3 provides an overview of the approach, which predicts the impact of M-APP on kids in two main phases. In the first phase, it preprocesses reviews of real mobile applications using NLP techniques. Each preprocessed review is then formatted using a BERT *Tokenizer* to pass into the BERT model for contextual word embeddings. Finally, the BERT model transforms each formatted review into contextual embedding vectors. In the second phase, the approach employs the contextual embedding vectors of the reviews and their corresponding category labels to train the A-CNN classifier. Subsequently, the trained classifier predicts the category of testing reviews based on their vectors. The proposed approach functions as follows:

1. It collects and labels the *HuggingFace* dataset of user reviews for M-APPs from https://huggingface.co/datasets/app_reviews/blob/main/app_reviews.py.
2. Next, it leverages NLP techniques for data preprocessing, which involves cleaning and formatting text data acquired from user reviews.
3. It formats the preprocessed textual data using BERT *Tokenizer* to transform it into a perceivable form by the BERT model to generate contextual word embeddings.
4. The feature extraction process involves converting formatted data into numerical representations, utilizing the BERT model for contextual word embeddings.
5. The proposed approach implements an A-CNN model using the extracted features as input for predicting the impact of M-APPs on kids. It proceeds to train the A-CNN model using 80% from the extracted features.
6. It assesses the performance results of the trained model on 20% test data, utilizing metrics like accuracy, precision, recall, and F1-score.

### 3.2 Illustrating example

The provided illustration is intended to demonstrate how the proposed approach is used to classify M-APP’s affects on children. Specifically, it involves a mobile app review for the package com.mill_e.twitterwrapper, which was gathered from the extracted dataset available on Hugging Face.

- **Review**: Good idea bad product I’m so frustrated! Half of my posts never actually get posted they just disappear!
- **Date**: April 13 2016
- **Class**: Negative

The subsequent sections further elaborate on the approach and explain its key steps.

### 3.3 Problem defination

The dataset comprises severity information regarding mobile apps and their corresponding details. When considering the collection of review reports for mobile apps denoted as *R*, each report *r* can be represented in the following manner:

$$\begin{array}{c} r=\;<t,i> \end{array}$$ (1)

where, *t* denotes the textual descriptions of review *r* and *i* demonstrates its impact on kids.

The proposed method categorizes the impact of M-APP on kids as either *Positive* or *Negative*. This categorization can be represented by the function *f*.

$$\begin{array}{c} f:r\to c \end{array}$$

$$\begin{array}{c} c\in \{Positive,Negative\},\;r\in R \end{array}$$ (2)

where *c* is the binary output of the classifier that can be either *Negative* or *Positive*.

### 3.4 Preprocessing

Preprocessing plays an important role in NLP tasks, as it prepares the raw text data for analysis and enhances the performance of M/DL models. By eliminating noise and addressing inconsistencies, preprocessing yields improved outcomes. Below are the key steps involved in preprocessing data that the proposed approach comprises.

- **Lowercase Conversion**: Lowercase text is a crucial initial step in NLP as it reduces inconsistencies, normalizes data, and enhances accuracy by unifying word representations for better semantic analysis and model performance.
- **Tokenization**: Tokenization divides text into smaller units (tokens), like words or sentences, essential for various tasks. The proposed approach utilizes word_tokenize() from NLTK for this purpose.
- **Spell correction**: In NLP, spell correction fixes wrong words, making text analysis better by fixing common spelling mistakes.
- **Stop-words Removal**: Getting rid of common words in NLP helps focus on important ones, though some tasks might need some of these words; the suggested method removes these words to make it easier for the BERT model and A-CNN classifier.
- **Stemming/Lemmatization**: Stemming shortens words using basic rules, sometimes creating non-real words, while lemmatization accurately reduces words to their base form considering context and part of speech in NLP.

After performing the preprocessing steps, the M-APP review *r* can be depicted as,

$$\begin{array}{c} r^{\prime }=\;<k_{j},i> \end{array}$$ (3)

$$\begin{array}{c} k_{j}=\;<k_{1},k_{2},\ldots ,k_{n}> \end{array}$$ (4)

where, *k*_1_, *k*_2_, …, *k*_*n*_ are the tokens generated after performing the preprocessing steps on the textual data of an M-APP review *r*.

### 3.5 BERT tokenizer input formatting

M-APP impact prediction on kids is closely associated with correctly representing essential textual elements to BERT and numerical representation to the A-CNN classifier. Recent research [35, 36] highlight diverse techniques for word embeddings in NLP, i.e., Word2Vec [36], GloVe [37], and FastText [38]. Yet, BERT [39] is a powerful NLP model trained on a lot of text and stands out for that reason. For a variety of tasks, it is effective since it can acquire rich contextual representations of words and phrases. The process of formatting input data outlines the steps for preparing data to generate BERT embeddings. Specifically, the “bert-base-uncased” variant of the “BertTokenizer” from the “Transformers” library is utilized. The process of formatting for review *r* is as follows:

- The *BertTokenizer* segments textual content from the M-APP review into a series of subword tokens. These subword units are converted to integer IDs through a predefined vocabulary. Additionally, the tokenizer introduces specialized tokens, namely *CLS* (Classification) and *SEP* (Separation), which represent the initiation and conclusion of the review description. The tokenization process can be presented in the following manner:
  $$\begin{array}{c} \text{S}=[\text{[CLS]}\;+\;{\text{Subw}}_{1}\;+\;{\text{Subw}}_{2}\;+\;\ldots \;+\;{\text{Subw}}_{n}\;+\;\text{[SEP]}] \end{array}$$ (5)
  where, *S* symbolizes the sequence of subwords within the M-APP review *r*′, Subw_*i*_ represents an individual subword, and *n* signifies the total count of subwords in a review *r*′.
- The BERT tokenizer utilizes a predefined vocabulary encompassing all conceivable tokens intelligible to a DL model. Identifiers such as “indices” are associated with each distinct token in this vocabulary. As the tokenized sequence, denoted as *S*, is prepared, each token within it is associated with its corresponding index as specified by the vocabulary of the BERT tokenizer.
  To illustrate, the BERT tokenizer’s vocabulary is assumed to encompass the subsequent tokens, accompanied by their respective index assignments:
  $$\begin{array}{cc} \text{[CLS]} & \to \text{index}\;\text{3001}\\ {\text{Subw}}_{1} & \to \text{index}\;\text{3002}\\ {\text{Subw}}_{2} & \to \text{index}\;\text{3003}\\ {\text{Subw}}_{3} & \to \text{index}\;\text{3004}\\ & \ldots \\ {\text{Subw}}_{n} & \to \text{index}\;\text{3050}\\ \text{[SEP]} & \to \text{index}\;\text{3051} \end{array}$$
  In essence, the vocabulary of the BERT tokenizer facilitates the smooth conversion of each token in a given sequence into its corresponding index. This index-based representation allows for efficient processing and comprehension by the model, enabling it to work with textual data effectively.
- The sequence is then transformed into input IDs. These input IDs form the ultimate input to the BERT model, which can be expressed as follows:
  $$\begin{array}{c} I=[101,{\text{id}}_{1},{\text{id}}_{2},\ldots ,{\text{id}}_{n},102] \end{array}$$ (6)
  In this representation, *I* denotes the sequence of input IDs, id_*i*_ signifies an individual input ID and *n* represents the count of IDs per M-APP review *r*. The resulting sequence *I* effectively encapsulates the tokenized textual content in a numerical format that the model can understand. This representation serves as input that the model processes to perform tasks, i.e., embedding generation, classification, sentiment analysis, or any other downstream task.
- Padding and Truncation involve preparing sentences for batch processing efficiency by ensuring consistency. In this stage, the IDs undergo adjustments to attain the highest allowable length for an M-APP review sequence. For the proposed approach, padding is applied when the total count of token IDs in a specific input sequence is shorter than the 512 tokens. This entails extending the sequence using ‘0’ until an allowable length is achieved. Conversely, if an input sequence exceeds 512 tokens, the excess tokens are eliminated to adhere to the prescribed length. This transformation can be expressed as follows:
  $$\begin{array}{c} I_{1:512}^{\prime }=\{\begin{array}{ll} I_{1:m}+{\left[\text{PAD}\right]}_{512-m} & \text{if}\;m<512\\ I_{1:512} & \text{if}\;m>512 \end{array} \end{array}$$
  where, $I_{1:512}^{\prime }$ signifies the conclusive input IDs after padding and truncation in the specific context of M-App review *r*. The variable *m* denotes the count of token IDs in the input sequence. This padding and truncation process ensures that input sequences are standardized to a consistent length, facilitating smooth processing within the BERT model.
- Attention masks serve to differentiate authentic tokens from padded ones within an input sequence. This distinction is pivotal as the transformer’s attention mechanism depends on these masks to give precedence to actual tokens while disregarding padded ones. These masks are binary, using 0s for padded tokens and 1s for genuine tokens. For instance, consider a sequence with a length of ‘n’, which is padded to a fixed number of ‘m’. Attention masks in this pattern are said to be [1, 1, …, 1, 0, 0, …, 0], where the first ‘n’ elements are 1s, indicating real tokens, while the remaining elements are 0s, indicating padded tokens. In matrix representation, the attention mask can be expressed as:
  $$M_{I^{\prime }}=[\begin{array}{cccc} 1 & 1 & \cdots & 1\\ 1 & 1 & \cdots & 1\\ \vdots & \vdots & \ddots & \vdots \\ 1 & 1 & \cdots & 1\\ 0 & 0 & \cdots & 0\\ \vdots & \vdots & \ddots & \vdots \\ 0 & 0 & \cdots & 0 \end{array}]$$
  This attention mask, denoted as *M*_*I*′_, corresponds to the input sequence *I*′. Attention masks ensure that the model effectively focuses on relevant tokens and disregards padding during processing, contributing to enhanced efficiency and meaningful analysis.

Using a sequence length of 32, the proposed approach formats text for the illustrating example presented in Section 3.2 as follows:

- **Input Text**: “Good idea bad product I’m so frustrated! Half of my posts never actually get posted they just disappear!”
- **Tokenization and Adding Special Tokens**: [[CLS] ‘Good’ ‘idea’ ‘bad’ ‘product’ ‘I’ “’” ‘m’ ‘so’ ‘frustrated’ ‘!’ ‘Half’ ‘of’ ‘my’ ‘posts’ ‘never’ ‘actually’ ‘get’ ‘posted’ ‘they’ ‘just’ ‘disappear’ ‘!’ [SEP]]
- **Token IDs**: [101, 2204, 2801, 2919, 4031, 1045, 1005, 1049, 2061, 10686, 999, 2431, 1997, 2026, 8461, 2196, 2941, 2131, 3947, 2027, 2074, 9451, 999, 102]
- **Padding and Truncation**: [101, 2204, 2801, 2919, 4031, 1045, 1005, 1049, 2061, 10686, 999, 2431, 1997, 2026, 8461, 2196, 2941, 2131, 3947, 2027, 2074, 9451, 999, 102, 0, 0, 0, 0, 0, 0]
- **Attention Masks**: [1, 1, 1, 1, 1, 1, 1, 1, 1, 1, 1, 1, 1, 1, 1, 1, 1, 1, 1, 1, 1, 1, 1, 1, 0, 0, 0, 0, 0, 0]

### 3.6 Contextual word embeddings calculation

The proposed method leverages the BERT-based-uncased word-piece model (downloaded for English from https://github.com/google-research/bert) developed by Google and pre-trained using data sourced from BookCorpus and Wikipedia corpora. The “Bert-base-uncased” model is used for calculating embeddings with default hyperparameters [39] on Google Colab. Depending on availability, Google Colab provides one free GPU (Tesla T4 or Tesla K80). Token IDs and attention masks are fed into the BERT model that calculates embeddings.

$$\begin{array}{c} E_{N\times 768}=BERT\left({\left(I^{\prime },M_{I^{\prime }}\right)}_{N\times 512}\right) \end{array}$$ (7)

In Eq 7, *E*_*N*×768_ represents the embedding matrix of size *N* × 768 for *N* projects (288065), *BERT* represents the BERT model, and (*I*′, *M*_*I*′_)_*N*×512_ is collection of padded and truncated token IDs and Attention masks of size *N* × 512. Finally, the resulting embeddings matrix can be represented as:

$$\begin{array}{c} E=[v_{1},v_{2},\ldots ,v_{N}] \end{array}$$ (8)

where, *E* is the embedding matrix with shape [288065, 768], and *v*_*i*_ is the embedding vector for the *i*-th M-APP review. The matrix *E* can then be used as a feature matrix for machine learning models. A pre-trained BERT model generates embedding vectors as shown in Table 1 for the example in Section 3.2.

**Table 1:** BERT embeddings for illustrating example.

Sentence	Token Ids	BERT Embeddings
Good idea bad product I’m so frustrated! Half of my posts never actually get posted they just disappear!	[101, 2204, 2801, 2919, 4031, 1045, 1005, 1049, 2061, 10686, 999, 2431, 1997, 2026, 8461, 2196, 2941, 2131, 3947, 2027, 2074, 9451, 999, 102]	[0.0021, -0.0086, 0.0098, -0.0062, 0.0042, 0.0011, -0.0023, 0.0077, 0.0055, -0.0099, 0.0088, -0.0043, 0.0027, -0.0015, 0.0039, 0.0061, -0.0074, -0.0012, 0.0008, 0.0093, 0.0085, -0.0056, 0.0024, 0.0023]

### 3.7 Proposed Attentional-Convolutional Neural Network Model

For M-APP impact prediction, this study employs an A-CNN model. The attention layer-based CNN model for binary text classification combines the strengths of both CNNs and attention mechanisms [40], resulting in a model that captures intricate patterns, long-term dependencies, and contextual meanings within text data. This sophisticated combination has demonstrated exceptional performance across a range of NLP tasks [41].

**Fig 4:**
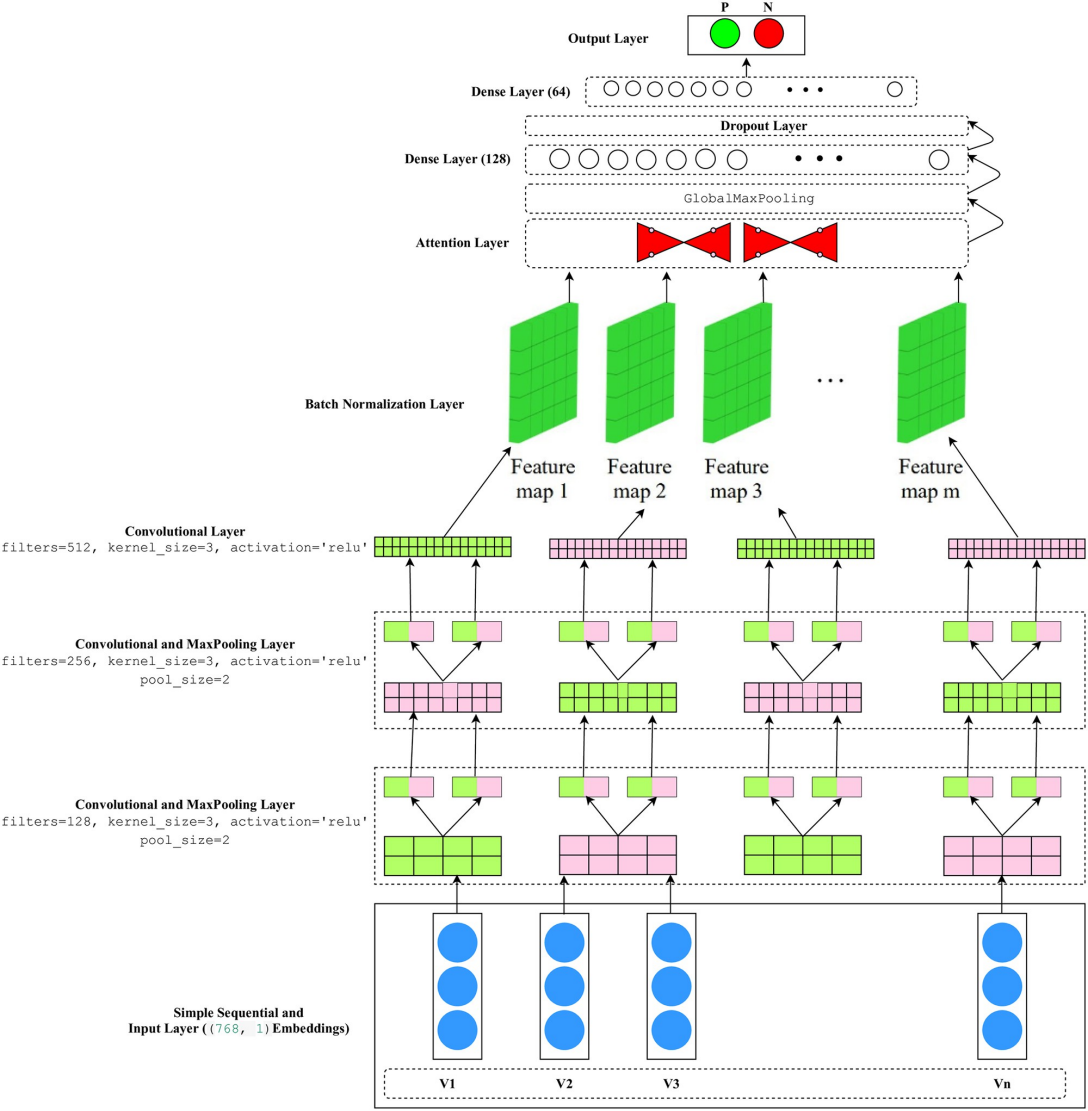
A-CNN framework.

The suggested approach designed a CNN model with an attention mechanism to capture intricate patterns and dependencies within the input data effectively. The model architecture, illustrated in Fig 4, consists of a series of carefully chosen layers to extract salient features from the BERT embeddings. The initial *Conv1D* layer employs a set of *128 filters* with a *kernel size* of *3* and employs the rectified linear unit (*ReLU*) activation function, effectively enhancing the non-linear transformations of the data and input_shape(768, 1). This is followed by a *MaxPooling1D* layer with a *pool size* of *2*, which downsamples the feature maps and reduces complexity. Subsequently, another *Conv1D* layer with *256 filters*, a *kernel size* of *3* and (*ReLU*) activation function is applied, further capturing intricate details from the data. A second *MaxPooling1D* layer is introduced to continue the downsampling process. The model then employs a *Conv1D* layer with *512 filters* and a *kernel size* of *3*, further increasing the receptive field and feature extraction capacity. To enhance the model’s stability and convergence, a *Batch Normalization* layer is added, normalizing the activations of the previous layer. The innovative inclusion of the *SeqSelfAttention* layer with a *sigmoid* activation function allows the model to learn attention-based relationships between different parts of the input sequence, focusing on crucial elements during feature extraction. The attention-enriched feature maps are pooled using a *GlobalMaxPooling1D* layer to capture the most salient features from each feature map. Two dense layers with *128* and *64* units, respectively, and *ReLU* activation functions enable the model to capture high-level patterns. A *Dropout* layer with a rate of *0.5* is leveraged to overcome overfitting. Finally, a single neuron with a *sigmoid* activation function is leveraged as the output layer for binary classification tasks. This architecture demonstrates the model’s ability to extract meaningful representations from BERT embeddings while exploiting attention-driven relationships to achieve better performance on the M-APP prediction. The model is trained and tested on the BERT embedding matrix *E*, employing an 80%-20% data splitting ratio.

## 4 Evaluation

To assess the model’s performance, research questions are formulated in this section. In the following sections, the collection of M-APP datasets as well as the process of constructing the proposed model are discussed. The next step is to establish the metrics and evaluate the results.

### 4.1 Research questions

The evaluation process accesses the proposed model by investigating the following research questions:

- **RQ1**: What is the performance of the proposed model compared with the SOTA approach?
- **RQ2**: What is the performance of the proposed model compared with other machine learning models?
- **RQ3**: What is the influence of the Attention-layer on the model’s performance?
- **RQ4**: How does BERT feature extraction differ from Word2Vec and FastText?
- **RQ5**: How does re-sampling effect?
- **RQ6**: How does preprocessing effect the proposed approach’s performance?

### 4.2 Dataset

**Fig 5:**
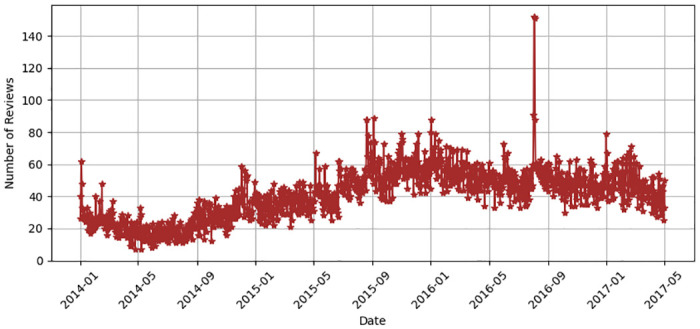
Dataset revfiew counts from 2014 to 2017.

**Fig 6:**
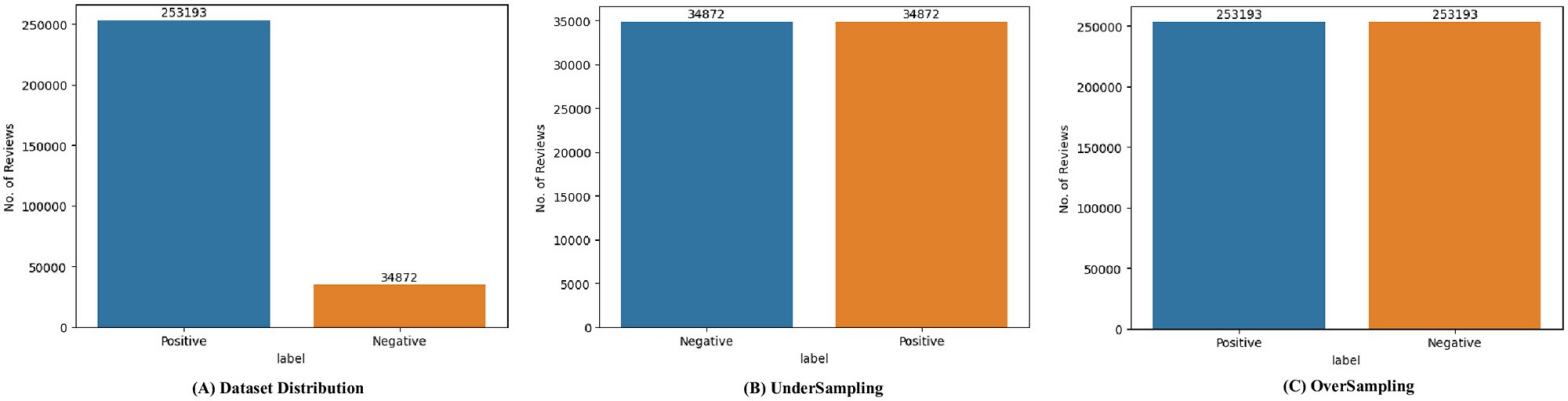
Dataset distribution with and without re-sampling.

**Fig 7:**
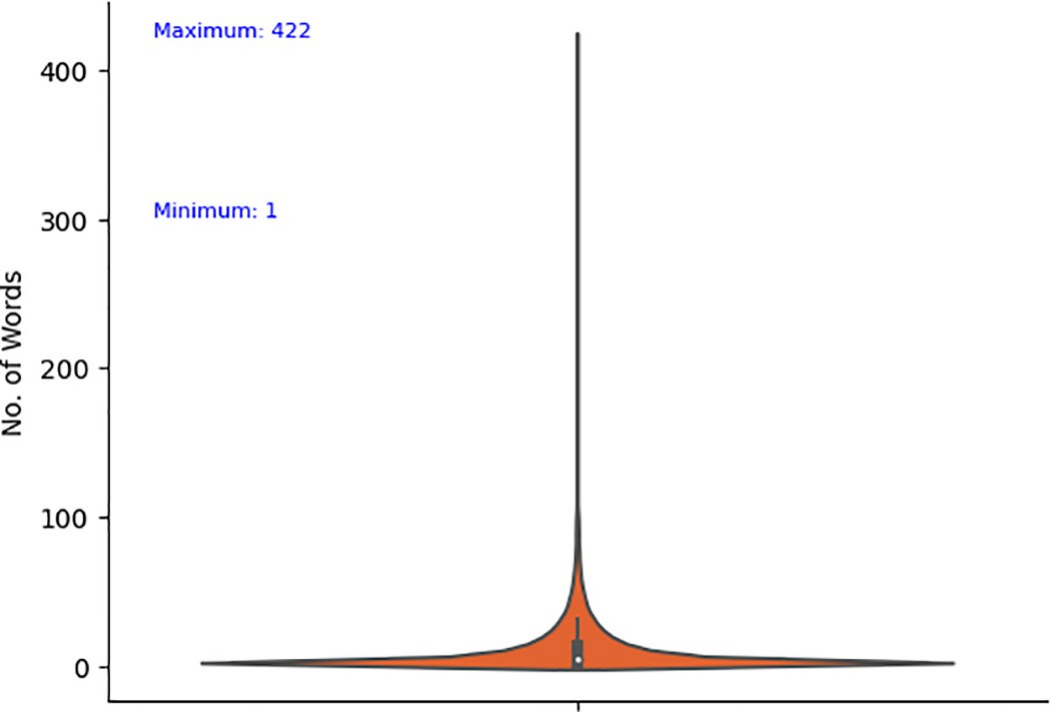
Minimum and maximum words in descriptions of M-APP reviews.

**Fig 8:**
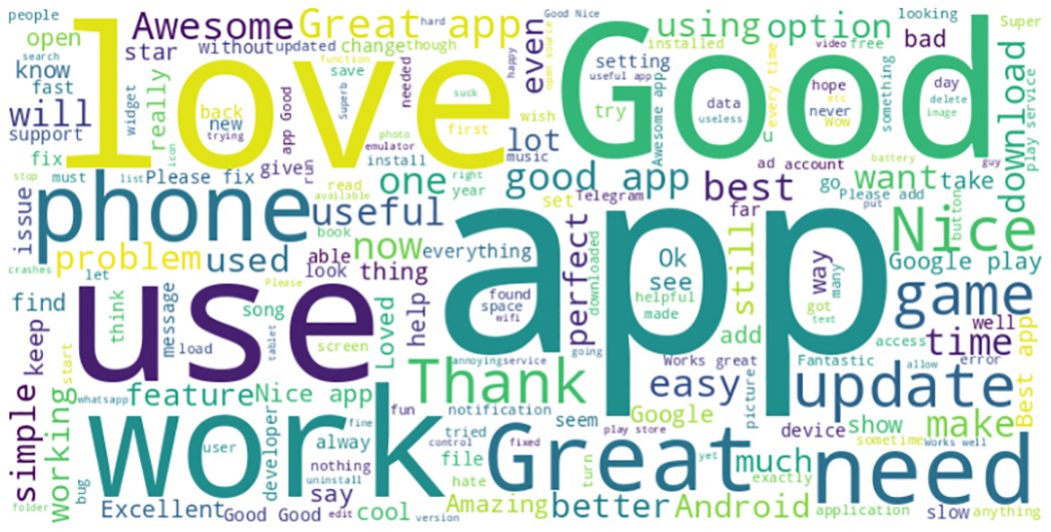
Words frequently used in M-App reviews.

The historical dataset of M-APP reviews has been obtained from the *HuggingFace* repository (https://huggingface.co/datasets) created by Giovanni Grano (University of Zurich), Sebastiano Panichella (University of Zurich), and Andrea di Sorbo (University of Sannio). This dataset is achieved by leveraging the *dataset’s* python library that returns messages in English. Notably, the data collection and analysis methods were conducted in strict accordance with the terms and conditions outlined by the data source. The process of extracting data involves collecting distinct attributes, i.e., package name, review content, date, and star rating (indicating severity) up to May 2017, as depicted in Fig 5. Table 2 summarizes the dataset’s statistics. It contains a substantial number of Android applications that are classified into 23 unique app categories. The dataset provides a summarized perspective on user feedback for these applications. It features an extensive collection of approximately 288,065 user reviews selected for use with the proposed model. A text-mining technique was used to extract these selected reviews. As indicated in Table 2, the chosen dataset comprises 288,065 entries, with each entry characterizing the degree of star rating of reviews as “1*,” “2*,” “3*,” “4*,” or “5*”. The proposed model labels the dataset by checking the reviews through a list of English negative check words. A review containing an English negative check word is labeled as *Negative*; otherwise, *Positive*. Among the total reviews, 34872 (12.11%) are labeled as *Negative* and 253193 (87.95%) are labeled as Positive as shown in Fig 6. Furthermore, Fig 7 shows that the review minimum count is 1 and the maximum count is 422. Fig 8 offers a rapid visual summary of the words or phrases that recurrently emerge in the text of app reviews. Within the word cloud, words that occur more frequently are depicted as larger and more visible, while words with lower frequency are depicted as smaller and less visible.

**Table 2:** Dataset statistics.

**Statistic**	**Count**	
M-App Reviews	288,065	
Categories	2	
**Class Distribution of Dataset**		
Negative	34872	12.107%
Positive	253193	87.953%
**Distribution of Star Ratings (Percentage)**		
1*	39984	13.878%
2*	13264	4.605%
3*	23196	8.051%
4*	37247	12.943%
5*	174374	60.523%

### 4.3 Evaluation metrices

To assess the performance of the A-CNN model, this study employs commonly used metrics [42–44] for binary classification, including accuracy, precision, recall, and F1 score as shown in Table 3.

**Table 3:** Evaluation metrics for classification.

Metric	Formula
Precision (P)	*TP*/(*TP* + *FP*)
Recall (R)	*TP*/(*TP* + *FN*)
F1 Score (F)	2*PR*/(*P* + *R*)
Accuracy (A)	(*TP* + *TN*)/(*TP* + *TN* + *FP* + *FN*)

where *TP* is counting the number of M-APP reviews that are correctly identified as *Positive*, *TN* is the count of reviews correctly labeled as *Negative*, *FP* counts the reviews that are mistakenly labeled as *Positive*, and *FN* identifies the reviews incorrectly marked as *Negative*.

### 4.4 Process

To assess the performance of the suggested approach, the following sequential actions are performed. Firstly, M-APP reviews denoted as *R* are extracted from the HuggingFace dataset. Secondly, the dataset is labeled as *Positive* or *Negative* based on a checklist of English negative words. Subsequently, this data undergoes preprocessing through NLP techniques. Next, the preprocessed dataset is passed to the BERT tokenizer for input formatting. Following input formatting, contextual word embeddings are calculated using the BERT-based-uncased model. Then dataset is split by the 80%-20% ratio. The training set encompasses 80% of the M-APP review embeddings, and the other 20% constitutes the testing set. Lastly, the A-CNN model is trained using the training samples, and the performance of this trained classifier is assessed on the testing samples. Steps to verify the effectiveness of the proposed model are:

- To commence, a collection of training embeddings is initially chosen.
- Following that, the training samples are employed to train the A-CNN Recurrent Neural Network (RNN), Long Short-Term Memory (LSTM), Logistic Regression (LR), Random Forest (RF), Gradient Boosting Classifier (GBC), Extreme Gradient Boosting classifier (XGB), Multinomial Naive Bayes (MNB), and Decision Tree (DT) models.
- In the following steps, the trained models are utilized to predict the impact of M-APPs based on the individual testing samples.
- The ultimate step involves evaluating each classifier’s accuracy, precision, recall, and F1 score to estimate its performance effectively.

### 4.5 Results

#### 4.5.1 Accuracy performance of A-CNN model

To address the first research question, the accuracy performance of the proposed approach is evaluated and compared with the state-of-the-art (SOTA) approach. The proposed model achieves an accuracy of 88.74% and improves recall of the SOTA approach by 0.32%. This is because attention mechanisms help CNNs concentrate on important parts and connections in the input data. This makes them good at finding complex patterns and far-reaching connections. Moreover, CNNs excel at detecting both local and global patterns through convolutional layers and pooling. Based on the analysis provided above, it is concluded that the attention layer helps the CNN model classify M-APPs for adolescents.

#### 4.5.2 Comparison of A-CNN with other classification models

To answer the second research question, this study performs a comparison of widely used text classification algorithms (LSTM, RNN, NN, XGB, LR, GBC, MNB, RF, DT) due to their competitive performance [45–47], where BERT embeddings are used as input features.

Tuning machine learning algorithm parameters is a practice commonly applied to statistical model fitting. In the case of the mentioned classification algorithms, the process involves training and assessing them using varying internal parameter configurations. This is all aimed at determining optimal parameter values. Additionally, a comparative analysis is conducted to evaluate how these classification algorithms perform under the influence of the most appropriate parameter settings and the most optimal parameter settings are considered.

Table 4 displays the evaluation results for the A-CNN, LSTM, RNN, XGB, LR, GBC, MNB, RF, and DT classifiers. The classifiers are listed in the first column. The accuracy, precision, recall, and F1 score metrics for LSTM, RNN, XGB, LR, GBC, MNB, RF, and DT are (88.74%, 88.91%, 99.61%, and 93.96%), (88.48%, 88.61%, 99.71%, and 93.83%), (87.93%, 87.93%, 99.71%, and 93.83%), (88.12%, 88.16%, 99.91%, and 93.66%), (88.06%, 88.56%, 99.24%, and 93.6%), (86.38%, 86.41%, 98.73%, 92.16%), (75.2%, 91.21%, 79.44%, and 84.92%), (61.84%, 52.23%, 61.84%, and 52.03%), and (48.05%, 26.2%, 26.32%, and 26.25%) respectively.

**Table 4:** Comparison of different classifiers with BERT embeddings (In%).

Model Name	Accuracy	Precision	Recall	F1 score
**A-CNN**	**88.74**	**88.91**	**99.61**	**93.96**
LSTM	88.48	88.61	99.71	93.83
RNN	87.93	87.93	99.98	93.57
XGB	88.12	88.16	99.91	93.66
LR	88.06	88.56	99.24	93.60
GBC	86.38	86.41	98.73	92.16
MNB	75.20	91.21	79.44	84.92
RF	61.84	52.23	61.84	52.03
DT	48.05	26.2	26.32	26.25

From Table 4, the following factors are observed:

- Proposed approach outperforms LSTM, RNN, XGB, LR, GBC, MNB, RF, and DT in accuracy, precision, and F1 score. The attentional CNN model surpasses LSTM, RNN, XGB, LR, GBC, MNB, RF, and DT models in specific contexts due to its unique architectural traits. Attention mechanisms help CNNs concentrate on important parts and connections in the input data. This makes them good at finding complex patterns and far-reaching connections. Moreover, CNNs excel at detecting both local and global patterns through convolutional layers and pooling.
- LSTM models closely trail behind CNNs, showing notable performance with high accuracy, precision, recall, and F1 scores of 88.48%, 88.61%, 99.71%, and 93.83% respectively. LSTM is good at understanding long-term patterns in data and fixing issues with gradients, and it works well for tasks like understanding languages and analyzing time series data.
- LSTM, RNN, NN, and GBC models show better recall than the Attentional CNN model because they’re good at dealing with uneven class numbers, understanding sequences (LSTM, RNN), handling complex connections between features (NN, GBC), and adjusting how complicated they are based on the data they’re working with. Attentional CNNs might miss some small but important things about specific classes. To resolve this issue, a CNN model is also implemented with the balanced dataset in Section 4.5.5 in which it outperforms other models.
- The Decision Tree model performs the worst among others, with lower accuracy, precision, recall, and F1 score. Its limitations lie in its simple structure, which makes it hard to understand complex connections in data. They also can’t handle complicated connections between features like XGB and RF do. Their sensitivity to changes in data makes them less reliable in noisy situations. Though simple, these limits show that more advanced models are better for tasks needing complex connections, sequences, and strong performance.

**Fig 9:**
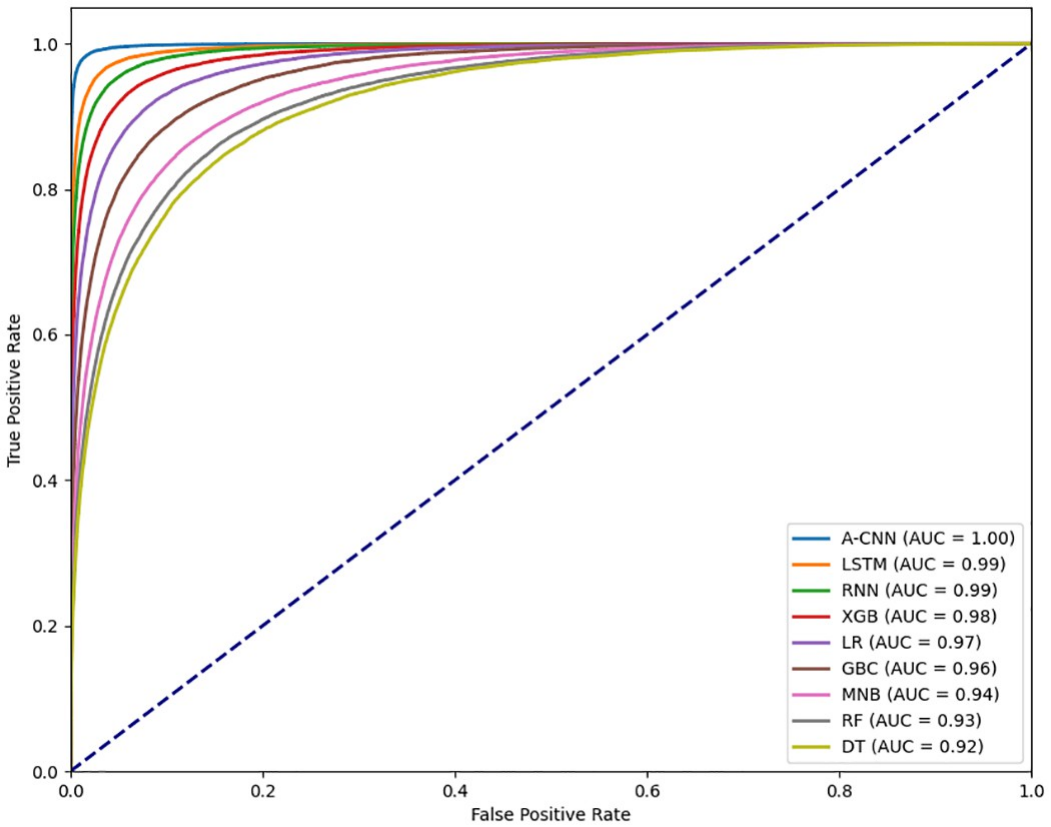
Receiver Operating Characteristic (ROC) curve.

The Receiver Operating Characteristic (ROC) curve (Fig 9) is also computed against each classification model to measure their performances. The true positive rate is plotted against the false positive rate for each model, with the diagonal blue dashed line representing random guessing. The legend indicates each model’s Area Under the Curve (AUC) value, with A-CNN having the highest at 1.00, indicating perfect classification. LSTM and RNN have AUCs of 0.99, XGB 0.98, LR 0.97, GBC 0.96, MNB 0.94, RF 0.93, and DT 0.92. The closer a curve is to the top left corner, the better the model performs. Overall, A-CNN is the best performer, while the other models also show strong classification abilities.

To provide a comprehensive comparison, additional experiments are conducted to measure the training time and computational resources required for the A-CNN model versus other models, i.e., LSTM, RNN, and SVM. All models were run on Google Colab, utilizing Tesla K80 GPUs, which provide access to substantial computational resources. While training time can vary due to internet connectivity and the availability of Google services, the results indicate that the A-CNN model requires more GPU hours compared to other machine and deep learning models. However, the A-CNN model remains competitive in terms of resource consumption, especially when considering the significant accuracy and performance improvements it offers.

Moreover, the inference time of each model is compared in a real-time environment to evaluate their feasibility for deployment on resource-constrained devices. The inference times for the A-CNN, LSTM, RNN, and LR models were approximately 0.050, 0.049, 0.049, and 0.041 milliseconds per sample, respectively, on a mobile device with a Qualcomm Snapdragon 865 processor. The findings suggest that although the A-CNN model is slightly more resource-intensive during inference compared to the LSTM and RNN models, it remains within acceptable limits for deployment on modern mobile devices. Additionally, the significant gains in accuracy, precision, and F1 score justify the slightly higher inference time.

Based on the above observations, It is concluded that A-CNN outperforms other machine learning models due to attention mechanisms that help CNNs concentrate on important parts and connections in the input data.

#### 4.5.3 Influence of attention layer in the proposed model

The proposed model is evaluated to assess the impact of the attention layer on responding to the third research question, the results are presented in Table 5. The table displays the influence of enabling and disabling the attention layer on the performance metrics of the model. For the “Enabled” status, the model achieved an accuracy of 88.74%, a precision of 88.91%, a recall of 99.65%, and an F1 score of 93.96%. Comparatively, with the attention layer “Disabled,” the model attained an accuracy of 88.69%, a precision of 88.99%, a recall of 99.43%, and an F1 score of 93.92%.

**Table 5:** Influence of attention layer (In%).

Status	Accuracy	Precision	Recall	F1 score
**Enabled**	**88.74**	**88.91**	**99.65**	**93.96**
Disabled	88.69	88.99	99.43	93.92

From Table 5 and Fig 4, the following factors are observed:

- Implementing the attention layer with CNN results in notable enhancements in accuracy, recall, and F1 score. Accuracy is increased by 0.06%, recall by 0.22%, and F1 score by 0.04%. Adding the attention layer made the model more complex, allowing it to weigh different parts of the data dynamically. This enhanced its focus and decision-making, offering advantages like selective attention, handling noise, and capturing fine details. Attention mechanisms help CNNs concentrate on important parts and connections in the input data.
- Specifically, the attention mechanism played a pivotal role in improving the model’s recall by 0.18%. By attending to the relevant features in the input data, the attention-equipped CNN model displayed a higher capability to correctly identify positive instances as shown in Fig 4, leading to an improved recall score.
- Removing the attention layer from A-CNN likely decreases precision because the model loses the ability to focus on and weigh the most relevant features of the input data, leading to a more uniform and less discriminative feature representation [48, 49]. This results in more false positives and a subsequent drop in precision.

Based on the analysis provided above, it is concluded that the incorporation of the attention layer into the A-CNN model has significant results and is a crucial component of the proposed approach.

#### 4.5.4 Impact of word embeddings with BERT

In addressing the fourth research question, a comparative analysis is conducted between BERT embeddings, FastText embeddings, and Word2Vec embeddings within the context of the A-CNN model. This investigation is illustrated in Table 6. When utilizing BERT embeddings, the A-CNN model achieves an accuracy of 88.74%, precision of 88.91%, recall of 99.61%, and F1 score of 93.96%. Comparatively, employing FastText embeddings results in A-CNN attaining an accuracy of 88.72%, precision of 88.76%, recall of 99.81%, and F1 score of 93.96%. Conversely, employing Word2Vec embeddings results in A-CNN attaining an accuracy of 87.93%, precision of 87.93%, recall of 100%, and F1 score of 93.58%.

**Table 6:** Impact of word embeddings with BERT (In%).

Feature	Accuracy	Precision	Recall	F1 score
**BERT**	**88.74**	**88.91**	**99.61**	**93.98**
FastText	88.72	88.76	99.81	93.96
Word2Vec	87.93	87.93	100	93.58

From Table 6, the following factors are observed:

- BERT embeddings outshine FastText embeddings in accuracy and precision by 0.023% and 0.17%. BERT understands words in sentences better because it looks at the whole sentence, unlike FastText which focuses more on smaller parts of words, missing some meanings. BERT is great at understanding complicated word connections and making language more detailed. It learns from lots of text, which helps it work better on different tasks. BERT can adjust its learning to new tasks, unlike FastText which needs to learn from scratch. BERT is good at handling new words by breaking them down into parts. Overall, because BERT understands words in context, it performs better at representing detailed language nuances compared to FastText.
- FastText embeddings outperform BERT embeddings in the recall by focusing on smaller word parts, improving recognition of shared patterns. FastText’s subword understanding aids pattern identification, and its context-independent nature enhances keyword recognition. FastText excels when the context is less crucial, with improved recall from targeted training data. BERT, on the other hand, focuses on context, which may affect recall.
- A-CNN improves Word2Vec embeddings by 0.9% accuracy, 1.22% precision, and 0.41% enhancement in F-measure by utilizing BERT word embeddings. BERT embeddings have a positive impact on the overall performance of the A-CNN model across various metrics, as demonstrated by these enhancements. A transformer-based deep neural network architecture is used in BERT, instead of a simple neural network used in Word2Vec for predicting word context. Its extensive pre-training with data from various sources allows it to understand word relationships and dependencies. Additionally, BERT’s bidirectional nature allows it to consider both the preceding and subsequent context around a word, in contrast to Word2Vec’s focus solely on preceding words. As a result, A-CNN (utilizing BERT embeddings) excels at predicting review severity compared to A-CNN (using Word2Vec embeddings).
- Recall with Word2Vec embeddings might outperform that with BERT embeddings due to Word2Vec’s focus on subword patterns, aiding recognition of shared prefixes/suffixes. This advantage helps capture specific variations of words, leading to better recall in certain contexts. BERT, although powerful, might sometimes struggle with such nuanced variations due to its contextual understanding.

Based on the analysis provided above, it is concluded that the incorporation of Bert embeddings into the A-CNN model has significant results and is an essential component of the proposed approach.

#### 4.5.5 Impact of re-sampling

To address the fifth research question, two distinct re-sampling methods are employed aimed at mitigating the class imbalance present within the dataset. These methods encompass both over-sampling and under-sampling techniques. In the context of over-sampling, the minority class is augmented by generating additional instances using the RandomOverSampler as shown in Fig 6. Conversely, the under-sampling approach involved rectifying imbalanced datasets by selectively removing surplus records from the majority class via the RandomUnderSampler. Evaluation encompasses both the original dataset and the resampled datasets, with outcomes detailed in Table 7. Specifically, oversampling reached an impressive accuracy of 91.45%, precision of 93.21%, recall of 99.98%, and the F1 score achieving values of 96.36%, respectively. The under-sampling method yielded values of 71.98%, 85.86%, 53.24%, and 65.73% for accuracy, precision, recall, and F1 score, respectively.

**Table 7:** Impact of re-sampling (In%).

Re-Sample	Accuracy	Precision	Recall	F1 score
Default	88.74	88.91	99.61	93.96
Over-Sampling	91.45	93.21	99.98	96.36
Under-Sampling	71.98	85.86	53.24	65.73

From Table 7, the following factors are observed:

- The utilization of the over-sampling approach led to significant improvements across multiple performance metrics, including accuracy, precision, recall, and F1 score. These enhancements can be attributed to the expanded and more balanced dataset that BERT was exposed to. This exposure facilitated a more effective grasp of meaningful patterns by the model. Through the oversampling technique, additional data has been incorporated and the overall performance of the model has been elevated.
- Alternatively, under-sampling reduces the count of samples in the class having majority instances, resulting in a loss of significant information. Consequently, when under-sampling is applied to the A-CNN model, both majority and minority classes exhibit declines.

Including Default, Over-Sampling, and Under-Sampling, distinct performance variations are observed in the form of accuracy, precision, recall, and F1-score. Moreover, the proposed approach with an over-sampled dataset has significant results. The significant impact of over-sampling, which increased accuracy by 19.47%, highlights the importance of addressing class imbalance in the dataset, as it enables the model to learn more effectively from a balanced representation of both classes. In contrast, preprocessing improved accuracy by 1.7% (reported in Section 4.5.6), word embeddings by 0.023% (reported in Section 4.5.4), and the attention layer by 0.06% (reported in Section 4.5.3), indicating that while these techniques fine-tune the model’s performance by enhancing data quality and representation, their effects are relatively minor compared to the foundational improvement achieved through resampling. This disparity underscores that balancing the training data is crucial for achieving substantial accuracy gains, while other methods contribute smaller but incremental benefits.

#### 4.5.6 Influence of preprocessings

Within the reviews of M-APPs, there exists irrelevant and meaningless information, such as punctuation marks and stop-words, as indicated in Section 3.4. Introducing such data to machine learning algorithms constitutes an unnecessary burden, leading to a decrease in their efficacy and an increase in computational resources. In addressing the sixth research question, a comparison of the suggested methodology is undertaken, both with preprocessing and without it. The assessment outcomes are detailed in Table 8, the accuracy, precision, recall, and F1 score of the proposed approach, with preprocessing enabled, are (88.74%, 88.91%, 99.61%, and 93.96%), whereas with preprocessing disabled, they are (87.04%, 87.21%, 98.22%, and 91.86%).

**Table 8:** Influence of preprocessing (In%).

Preprocessing	Accuracy	Precision	Recall	F1 score
Enabled	88.74	88.91	99.61	93.96
Disabled	87.04	87.21	98.22	91.86

Here are some observations based on Table 8:

- The introduced methodology, coupled with preprocessing layers, achieves noticeable advancements in its performance. The evaluation outcomes indicate that there is a performance enhancement of 1.95% in accuracy, 1.95% in precision, 1.41% in recall, and 2.29% in the F1 score.
- The proposed method’s performance drops significantly without preprocessing. This might happen because without preprocessing, the proposed approach might end up including extra words (like common words or words in their original form) that don’t help much. This could make the method slower and less effective.

Based on the analysis provided above, it is concluded that the incorporation of deep preprocessing layers for the textual information of the M-APP reviews is a crucial component of the proposed approach.

## 5 Conclusion and future work

The paper discusses the importance of accurately classifying M-APP reviews to protect adolescents from inappropriate content, especially with the advent of 6G technology. It exploits a deep learning model, incorporating NLP techniques and BERT embeddings, to predict the potential negative impact of M-APPs on adolescents. It uses the 80%:20% validation technique to evaluate the proposed A-CNN model. The model achieves high accuracy, precision, recall, and F1-score, outperforming other classifiers. In the future, I intend to explore the scalability of the model to handle a larger volume of M-APP reviews, as the app ecosystem is continuously growing. I also would like to incorporate not only textual features but also multimedia elements, i.e., images and videos within app reviews for a more comprehensive assessment of content. This would require research in multimodal deep learning. By addressing these aspects, future research can further enhance the capabilities and ethical considerations of content classification systems for M-APP reviews, ensuring better protection for adolescents in the ever-changing landscape of mobile technology.

## Supporting information

S1 Dataset Exploited dataset for the evaluation of the proposed approach. (ZIP)
