# Supplementary material for: Automatic classification of mobile apps to ensure safe usage for adolescents
Source: PLoS One. 2025 Jan 16;20(1):e0313953. doi: 10.1371/journal.pone.0313953 (PMC11737711; doi:10.1371/journal.pone.0313953)
Supplement: S1 Dataset — (ZIP) [file pone.0313953.s001.zip › sealuzh_app_reviews · Datasets at Hugging Face - WebPage.html]

sealuzh/app\_reviews · Datasets at Hugging Face


Hugging Face

- Models
- Datasets
- Spaces
- Posts
- Docs
- Solutions
- Pricing
- ---

Hugging Face is way more fun with friends and colleagues! 🤗
Join an organization

Dismiss this message

# Datasets: sealuzh / app\_reviews like 24

Tasks: 

Text Classification

Modalities: 

Text

Formats: 

parquet

Sub-tasks: 

text-scoring

sentiment-scoring

Languages: 

English

Size: 

100K - 1M

Libraries: 

Datasets

pandas

Croissant

+ 1

License:

unknown

Dataset card  Viewer  Files Files and versions  Community

4

Dataset Viewer

 Auto-converted to Parquet API Embed 

View in Dataset Viewer

Viewer

Subset (1)

default · 288k rows

default (288k rows) 

Split (1)

train · 288k rows

train (288k rows)

  

| package\_name  stringclasses  395 values | review  stringlengths  1  2.34k | date  stringlengths  11  17 | star  int8  1  5 |
| --- | --- | --- | --- |
| com.mantz\_it.rfanalyzer | Great app! The new version now works on my Bravia Android TV which is great as it's right by my rooftop aerial cable. The scan feature would be useful...any ETA on when this will be available? Also the option to import a list of bookmarks e.g. from a simple properties file would be useful. | October 12 2016 | 4 |
| com.mantz\_it.rfanalyzer | Great It's not fully optimised and has some issues with crashing but still a nice app especially considering the price and it's open source. | August 23 2016 | 4 |
| com.mantz\_it.rfanalyzer | Works on a Nexus 6p I'm still messing around with my hackrf but it works with my Nexus 6p Trond usb-c to usb host adapter. Thanks! | August 04 2016 | 5 |
| com.mantz\_it.rfanalyzer | The bandwidth seemed to be limited to maximum 2 MHz or so. I tried to increase the bandwidth but not possible. I purchased this is because one of the pictures in the advertisement showed the 2.4GHz band with around 10MHz or more bandwidth. Is it not possible to increase the bandwidth? If not it is just the same performance as other free APPs. | July 25 2016 | 3 |
| com.mantz\_it.rfanalyzer | Works well with my Hackrf Hopefully new updates will arrive for extra functions | July 22 2016 | 5 |
| com.mantz\_it.rfanalyzer | Good job Tried a few . This has to be the most stable and best to my liking . And a whole world of settings to play with. However we are quite limited on frequency band width id like to see it lowered to 14.mhz if possible | July 19 2016 | 5 |
| com.mantz\_it.rfanalyzer | Working great on Nexus6 | July 05 2016 | 5 |
| com.mantz\_it.rfanalyzer | Works with RTL and Nextbook Aries 8. Demod stops working if the scan width is changed requiring restart. | May 19 2016 | 5 |
| com.mantz\_it.rfanalyzer | Works with RTL SDR Works but no audio when demodulating | April 24 2016 | 3 |
| com.mantz\_it.rfanalyzer | Awsome App! Easy to use works great on Notes w / Realtek dongle. | April 16 2016 | 5 |
| com.mantz\_it.rfanalyzer | I'll forgo the refund. But no go with Watson dongle... Nexus 9. Yet to try on Nexus 6p. So very disappointed!!! :-( | March 31 2016 | 1 |
| com.mantz\_it.rfanalyzer | looks like a great program 1 of its kind I don't have the necessary hardware to utilize it though. | March 30 2016 | 4 |
| com.mantz\_it.rfanalyzer | This thing is great. I am at the bottom of the learning curve but in the few minutes I have played with it it seems like it will be easy to learn and to use. Just what I hoped for. | February 27 2016 | 5 |
| com.mantz\_it.rfanalyzer | Crash when set a demodulation! :( | February 22 2016 | 2 |
| com.mantz\_it.rfanalyzer | Good Good | February 22 2016 | 5 |
| com.mantz\_it.rfanalyzer | Support for sdrplay This software works fine on rtl sdr upconverter.But doesn't support sdrplay.Hope you add this support in near future. | February 20 2016 | 4 |
| com.mantz\_it.rfanalyzer | Nice Tested with Rtl SDR. Working fine although I saw weird behavior around 2.4Ghz. Not sure if this is the app. What is badly missing is some kind of bookmarks/favourites feature. | February 20 2016 | 4 |
| com.mantz\_it.rfanalyzer | doesn't work with rtl-2832 Says it works with rtl devices but doesn't. never displays anything even though the rtl 2832 works with other spectrum analyzers. no help files anywhere so good luck figuring out what's wrong! | February 10 2016 | 1 |
| com.mantz\_it.rfanalyzer | Sweet Pick your source device in settings. Default is hackrf. May need driver as well | February 01 2016 | 5 |
| com.mantz\_it.rfanalyzer | Great app! Works really well with Galaxy note 5 | February 01 2016 | 5 |
| com.mantz\_it.rfanalyzer | The app looks good but does not work so far with lenovo a936 kitkat. the sdr might draw to much current. Tried a spit cable for extra power but it did not help so far. | January 31 2016 | 4 |
| com.mantz\_it.rfanalyzer | Works great I am using HackRF One with this apps. | January 29 2016 | 5 |
| com.mantz\_it.rfanalyzer | A God send! Awful interference problem with my 868MHz central heating system. 20min with this app and an rtlsdr dongle tracked the problem to an old controller with a near flat battery transmitting a continuous unmodulated carrier. | January 09 2016 | 5 |
| com.mantz\_it.rfanalyzer | App Great but This app is great but the only thing that's missing is its ability to store frequencies and presets like the competing app. | December 13 2015 | 4 |
| com.mantz\_it.rfanalyzer | awesome app seems stable and runs well on Note 4 with RLT-SDR can't wait to try it out on the HackRF One. Happy to support the developer! | November 24 2015 | 5 |
| com.mantz\_it.rfanalyzer | Simple and perfect About this software rtl sdr is very useful ... installed done. Thanks. | November 08 2015 | 5 |
| com.mantz\_it.rfanalyzer | Samsung note 3 neo ok | October 15 2015 | 5 |
| com.mantz\_it.rfanalyzer | This works great so far. Any plans for support of Q branch direct sampling for HF reception on RTL SDR? Maybe the option is there and I missed it. Great app keep up the great work. | October 12 2015 | 5 |
| com.mantz\_it.rfanalyzer | Nice Work Wish it could transmit too on Hack | October 04 2015 | 5 |
| com.mantz\_it.rfanalyzer | Great application Work's well with my ZenFone 5 | October 03 2015 | 5 |
| com.mantz\_it.rfanalyzer | Very nice. Great UI works like a charm. If you have an NVIDIA shield try spamming the connect/play button. | September 25 2015 | 5 |
| com.mantz\_it.rfanalyzer | Additional info Used it w/RTL-SDR (R820T) on my phone (Moto G) also and it works great there too. I already had FM on this phone and now have NWS taxis Aviation Marine etc. Hopefully scanner update soon. Love it! Have it also on my new Chuwi HI8 dual-mode tablet so you can add that to the list | September 25 2015 | 5 |
| com.mantz\_it.rfanalyzer | Fun app - A little tedious though I enjoy using this app but can be a bit tedious trying to get everything set and configured just right. Once your SDR dongle has been properly configured you can then enjoy using it. Nice little app :&gt;) | September 24 2015 | 4 |
| com.mantz\_it.rfanalyzer | Works Great. Works great on a 2015 Medion Lifetab S10346 (MD99282). 10.1"""""""""""""""". Android 5. Watson wideband 100Khz-2Ghz model W-SDRX1 rtl dongle based receiver. Mostly Amatuer Band and Airband use. HF and VHF-UHF receive is fine if you cn feed it from any half decent antenna farm even works ok with indoor antenna systems with a bit more interference. It is possible to power the receiver from the tablet with the supplied usb otg cable but a otg cable with power in capablity is highly recommended. Great app works as good as any windows linux or mac sdr apps that I own. Fine Business to the developer. Wish that I could rate your app with more than 5 stars makes it possible for me to go portable or mobile with the sdr without having to carry a laptop everywhere."" | September 03 2015 | 5 |
| com.mantz\_it.rfanalyzer | Good Works well on Oneplus One with and without a powered otg cable | August 23 2015 | 5 |
| com.mantz\_it.rfanalyzer | Works great with rtl Sdr and HTC one x Thanks very much. | August 18 2015 | 5 |
| com.mantz\_it.rfanalyzer | Amazing A very useful app. I originally downloaded the free version from github but decided to buy it here as the dev deserves the money. | August 10 2015 | 5 |
| com.mantz\_it.rfanalyzer | Works well but would like added features The main additional feature I would like is a way to be able to adjust the spec an amplitude reference so that I can calibrate it to dBm. | August 05 2015 | 4 |
| com.mantz\_it.rfanalyzer | Do I need to hack my phone for this application to work | August 01 2015 | 1 |
| com.mantz\_it.rfanalyzer | Works on Motorola Droid Mini. Very cool app. I would like to try and DF power line noise using this app coupled with the RTL-SDR dongle. nice job on the app! | July 16 2015 | 5 |
| com.mantz\_it.rfanalyzer | The most fun you can have with a dongle ;-) Works perfectly with my Oneplus One and a generic RTL-SDR dongle. Well worth paying a few pence for to support the author. | June 18 2015 | 5 |
| com.mantz\_it.rfanalyzer | Does not work Did not work with my rtl-sdr | May 23 2015 | 1 |
| com.mantz\_it.rfanalyzer | Works ok Please improve tuning... Add possibility to tune like PC SDR software by flipping digit up/down. Currently it's too many taps to fine-tune | May 23 2015 | 4 |
| com.mantz\_it.rfanalyzer | Great app! Works flawlessly on my Samsung galaxy S5. | April 27 2015 | 5 |
| com.mantz\_it.rfanalyzer | Works great. Thanks! | April 25 2015 | 5 |
| com.mantz\_it.rfanalyzer | Great app Works well | April 21 2015 | 5 |
| com.mantz\_it.rfanalyzer | Working great with RTL Great job! This is working great with my RTL SDR. Well worth the money! Keep up the great work dev! | April 17 2015 | 5 |
| com.mantz\_it.rfanalyzer | Cool App Works great on my Galaxy Note 4 and Galaxy Tab Pro with my HackRF One. I've not yet tested with my RTL-SDR.. | April 17 2015 | 5 |
| com.mantz\_it.rfanalyzer | How do I change sample on the app rate I am using a remote server | April 08 2015 | 3 |
| com.mantz\_it.rfanalyzer | Works on Nexus 9 Perfectly works on Nexus 9 with RTL-SDR! | April 02 2015 | 5 |
| com.mantz\_it.rfanalyzer | Great App - Snags with Kitkat Worked fine on Jellybean but started throwing snags after upgrade. Will try logging and send it to the Author... | March 30 2015 | 4 |
| com.mantz\_it.rfanalyzer | Awesome and epic This application is awesome. This worked with my USB dongle. | March 29 2015 | 5 |
| com.mantz\_it.rfanalyzer | Really cool gadget for RF engineers Restarts driver when changing orientation. Please fix that. Additionally would like to have MUTE button alongside with other buttons. Another issue is: let user choose whether he/she wants kHz MHz or GHz when entering freq. Thanks in advance! | March 28 2015 | 5 |
| com.mantz\_it.rfanalyzer | Very useful Works as shown | March 24 2015 | 5 |
| com.mantz\_it.rfanalyzer | Awesome work - thanks for open sourcing! So happy to discover this project. Thank you so much for the first GPL open source Android sdr spectrum explorer I've seen. Very exciting. It's also great that you've made it easy to put a $ in the tip jar via the app store. I'm really jazzed about RF Explorer! Keep up the great work! Other features it would be wonderful to see in future versions (these would be icing on the cake!): - DC offset correction - Audio gain control - (maybe even audio spectrum display?) | March 21 2015 | 5 |
| com.mantz\_it.rfanalyzer | Amazing!!! Works like a charm! Nice feature to save recordings into file. Would be even better to transmit from file on HackRF! 73 SQ7MHZ | March 20 2015 | 5 |
| com.mantz\_it.rfanalyzer | Xperia Z Tablet Grabbed the app to play around with RTL SDR. Better signal clarity than SDR# on my laptop. Very happy so far with $15 total investment. Dongle powered up straight from the tablet. Don't have a HackRF to test. | March 11 2015 | 5 |
| com.mantz\_it.rfanalyzer | Awesome. Works well on Amiko A3 OnePlus One/CM11 using RTL-SDR. Bargain price! | March 10 2015 | 5 |
| com.mantz\_it.rfanalyzer | Great little analyzer and demodulator! | February 05 2015 | 5 |
| com.mantz\_it.rfanalyzer | The real deal Outstanding | January 25 2015 | 5 |
| com.mantz\_it.rfanalyzer | Pretty cool Reset the driver/itself when going from landscape to portrait and vice versa | January 24 2015 | 4 |
| com.mantz\_it.rfanalyzer | Works ok have a bug it seems switching modes am to fm the sound disappears if u restart the app sound is back until i change modes again. I9505 on kitkat. Other than that its a usefull app. | January 23 2015 | 4 |
| com.mantz\_it.rfanalyzer | Amazing Love the added demos now. Using mine with hackrf. Also tested with RTL. Both work perfectly! Thanks for a great implementation. | January 20 2015 | 5 |
| com.mantz\_it.rfanalyzer | Excellent job This app works great. No head aches no yelling. I followed the instructions downloaded the driver. My rtlsdr worked not a single problem. I can't get any rtlsdr to work on my desk tops. Thank you oh great one! | January 19 2015 | 5 |
| com.mantz\_it.rfanalyzer | Works Great App works well with rtl-sdr usb dongle except that it has a much more restricted freq range than a competitors app with the same dongle. Possibly a user error. Ie can not tune below 24Mhz. Get out of range error. Hoping this is user error as the support and upgrade path from this dev appears to be much better than competitors app. UPDATE: After response from Dev. it appears that the app is correct the tuner in my dongle has a24MHz lower limit The competitors app appears to tune much lower ( doesn't throw error and has signals) but it is not actually tuning lower. After purchase support for this app is superb. Can't wait for future updates. Thank You | January 19 2015 | 3 |
| com.mantz\_it.rfanalyzer | Excellent app Works great on my Note 8.0 and with a powered micro-usb-hub works on my Z3 compact too. Looking forward to scanning and would love to see audio recording added. Thanks :-) | January 07 2015 | 5 |
| com.mantz\_it.rfanalyzer | Nexus 10 works well Great app works well on my Nexus 10. | January 07 2015 | 5 |
| com.mantz\_it.rfanalyzer | Superb on Tesco Hudl 2 Worked first time with RTL dongle and pre-installed driver from Martin Marinov | January 01 2015 | 5 |
| com.mantz\_it.rfanalyzer | Definitely worth $0.99 Only $0.99! Since the developer is quickly listening to user feedback - this app is probably going to be better than SDR Touch very soon! I have two wishes - Material Design and remove the screen orientation settings to use Android's settings. Update: one of those wishes has already come true! The logo and name of the app is missing from the top right corner though. | December 10 2014 | 5 |
| com.mantz\_it.rfanalyzer | Awesome app Just what I've been looking for. | December 03 2014 | 5 |
| com.mantz\_it.rfanalyzer | BLU life xl works | November 23 2016 | 5 |
| com.mantz\_it.rfanalyzer | Not working Not working at oppo. Saying source not available for hr | November 23 2016 | 1 |
| com.mantz\_it.rfanalyzer | Perfect! What a brilliant app for the price. Works perfectly on my Oneplus One with RTL-SDR dongle and otg cable. | December 05 2016 | 5 |
| com.mantz\_it.rfanalyzer | Works Motorola g4 plus and rtlsdr | December 09 2016 | 4 |
| com.mantz\_it.rfanalyzer | Unable to set sampling rate on infinix X2 with MT6753 Unable to set sampling rate on the hackrf one on phone with MT6753 processor. | December 13 2016 | 3 |
| com.mantz\_it.rfanalyzer | Crashes with in 10 seconds I cant even use this for more then 15 seconds | December 25 2016 | 1 |
| com.mantz\_it.rfanalyzer | Squelch is non-existent in this app. The developer claims it exists but it is impossible to set up. No matter how I adjust the gain I always hear the RF noise in between the transmissions. This app will make me deaf. The only way to eliminate the noise is to manually move the dB slider up and down which very impractical and annoying. Every free PC SDR software has perfectly working squelch. Why this one doesn't? Could be a great app but luck of squelch makes it unusable. I would expect this to be fixed. | February 12 2017 | 2 |
| com.mantz\_it.rfanalyzer | Works well on Huawei Mediapad ML-802L and RTL | February 12 2017 | 5 |
| com.mantz\_it.rfanalyzer | For €0.79 this app is 10x less expensive than sdr touch and for that reason I gave it four stars. There are some improvements needed such as setting squelch and bandwidth could be easier recording audio as well as the entire band there is a high-pitched tone when using wide band fm a way that makes it easier to fine tune and stability improvements. This app works pretty well on my device (OnePlus 2) but it does crash every now and then (when changing between portrait and landscape for example). I think this app is great for the price and does pretty much everything I need but I would like to see these improvements. | February 20 2017 | 4 |
| com.mantz\_it.rfanalyzer | I think it would be neat if there were to be a wifi analysis tool built in which would overlay a spectrum analysis with access points and channels. | March 14 2017 | 5 |
| rs.pedjaapps.alogcatroot.app | A-Z 8apple | August 01 2016 | 5 |
| rs.pedjaapps.alogcatroot.app | Logcat Its good help | July 30 2016 | 1 |
| rs.pedjaapps.alogcatroot.app | Great logging app Very helpful finding issues within apps or the OS or even extra mods! Props | March 09 2016 | 5 |
| rs.pedjaapps.alogcatroot.app | Great app | February 25 2016 | 4 |
| rs.pedjaapps.alogcatroot.app | Crude compared to catlog | February 21 2016 | 3 |
| rs.pedjaapps.alogcatroot.app | Best of logcat Not Working on MM. Please fix this. Thanks.. | January 20 2016 | 5 |
| rs.pedjaapps.alogcatroot.app | LOL @Video Crew this is a LOG VIEWER and is unable to delete any settings from your phone. | December 12 2015 | 5 |
| rs.pedjaapps.alogcatroot.app | Unresponsive maybe some threads are in order. Logcat is better foss | August 29 2015 | 2 |
| rs.pedjaapps.alogcatroot.app | This isnt work at all! | June 25 2015 | 1 |
| rs.pedjaapps.alogcatroot.app | great | April 16 2015 | 5 |
| rs.pedjaapps.alogcatroot.app | Internet permission Unnecessary internet permission | October 17 2014 | 1 |
| rs.pedjaapps.alogcatroot.app | Works well and looks pretty good. | October 13 2014 | 5 |
| rs.pedjaapps.alogcatroot.app | You cant use Android without even debugging it You cant use Android without even debugging it could you? Helps tracking down bugs. | December 01 2016 | 5 |
| rs.pedjaapps.alogcatroot.app | Good | November 29 2016 | 5 |
| rs.pedjaapps.alogcatroot.app | Nothing worked. Blank screen. | December 19 2016 | 1 |
| rs.pedjaapps.alogcatroot.app | Description about Internet access is a lie. This app connects to the Internet even at its first start doing many connections to Amazon servers. And also doesn't show any log not even its own log without root. Another bad XDA sponsored project. | January 17 2017 | 1 |
| rs.pedjaapps.alogcatroot.app | It is very helpful | March 29 2017 | 5 |
| com.iskrembilen.quasseldroid | Crippling power consumption The devs really need to work on the power consumption. This app destroys my battery. Other than that it's really hard to give up quassel when you live and breath IRC. | October 27 2016 | 4 |
| com.iskrembilen.quasseldroid | Unusable in large rooms No way to filter out joins or parts. | October 23 2016 | 2 |
| com.iskrembilen.quasseldroid | is there a way to hide channel list when using a tablet? | September 27 2016 | 3 |

End of preview. Expand
in Dataset Viewer.

---

 

- Previous
- 1
- 2
- 3
- ...
- 2,881
- Next

- Dataset Structure
  - Data Instances
  - Data Fields
  - Data Splits
- Dataset Creation
  - Curation Rationale
  - Source Data
  - Annotations
  - Personal and Sensitive Information
- Considerations for Using the Data
  - Social Impact of Dataset
  - Discussion of Biases
  - Other Known Limitations
- Additional Information
  - Dataset Curators
  - Licensing Information
  - Citation Information
  - Contributions

# Dataset Card for [Dataset Name]

### Dataset Summary

It is a large dataset of Android applications belonging to 23 differentapps categories, which provides an overview of the types of feedback users report on the apps and documents the evolution of the related code metrics. The dataset contains about 395 applications of the F-Droid repository, including around 600 versions, 280,000 user reviews (extracted with specific text mining approaches)

### Supported Tasks and Leaderboards

The dataset we provide comprises 395 different apps from F-Droid repository, including code quality indicators of 629 versions of these
apps. It also encloses app reviews related to each of these versions, which have been automatically categorized classifying types of user feedback from a software maintenance and evolution perspective.

### Languages

The dataset is a monolingual dataset which has the messages English.

## Dataset Structure

### Data Instances

The dataset consists of a message in English.

{'package\_name': 'com.mantz\_it.rfanalyzer',
'review': "Great app! The new version now works on my Bravia Android TV which is great as it's right by my rooftop aerial cable. The scan feature would be useful...any ETA on when this will be available? Also the option to import a list of bookmarks e.g. from a simple properties file would be useful.",
'date': 'October 12 2016',
'star': 4}

### Data Fields

- package\_name : Name of the Software Application Package
- review : Message of the user
- date : date when the user posted the review
- star : rating provied by the user for the application

### Data Splits

There is training data, with a total of : 288065

## Dataset Creation

### Curation Rationale

[More Information Needed]

### Source Data

#### Initial Data Collection and Normalization

[More Information Needed]

#### Who are the source language producers?

[More Information Needed]

### Annotations

#### Annotation process

[More Information Needed]

#### Who are the annotators?

[More Information Needed]

### Personal and Sensitive Information

[More Information Needed]

## Considerations for Using the Data

### Social Impact of Dataset

With the help of this dataset one can try to understand more about software applications and what are the views and opinions of the users about them. This helps to understand more about which type of software applications are prefeered by the users and how do these applications facilitate the user to help them solve their problems and issues.

### Discussion of Biases

The reviews are only for applications which are in the open-source software applications, the other sectors have not been considered here

### Other Known Limitations

[More Information Needed]

## Additional Information

### Dataset Curators

Giovanni Grano - (University of Zurich), Sebastiano Panichella - (University of Zurich), Andrea di Sorbo - (University of Sannio)

### Licensing Information

[More Information Needed]

### Citation Information

@InProceedings{Zurich Open Repository and
Archive:dataset,
title = {Software Applications User Reviews},
authors={Grano, Giovanni; Di Sorbo, Andrea; Mercaldo, Francesco; Visaggio, Corrado A; Canfora, Gerardo;
Panichella, Sebastiano},
year={2017}
}

### Contributions

Thanks to @darshan-gandhi for adding this dataset.

Downloads last month
:   115

Use this dataset

Edit dataset card

Homepage:

Home Page

Repository:

Repo Link

Paper:

Link

\*\*Leaderboard:

\*\*Leaderboard:

Point of Contact:

Darshan Gandhi

Size of downloaded dataset files:

13.2 MB

Size of the auto-converted Parquet files:

13.2 MB

Number of rows:

288,065

## Models trained or fine-tuned on sealuzh/app\_reviews

#### tasksource/deberta-small-long-nli

Zero-Shot Classification
• 
Updated
1 day ago
• 

12.4M
• 

23

#### sileod/deberta-v3-base-tasksource-nli

Zero-Shot Classification
• 
Updated
1 day ago
• 

20.4k
• 

115

#### sileod/deberta-v3-large-tasksource-nli

Zero-Shot Classification
• 
Updated
Feb 17
• 

9.91k
• 

29

#### tasksource/deberta-base-long-nli

Zero-Shot Classification
• 
Updated
1 day ago
• 

4.41k
• 

15

#### sileod/deberta-v3-xsmall-tasksource-nli

Zero-Shot Classification
• 
Updated
Jul 4
• 

176
• 

3

#### Yanni8/star-predictor-v0.1

Text Classification
• 
Updated
Nov 28, 2023
• 

9
• 

1

Browse 9 models trained on this dataset


Company

© Hugging Face

TOS
Privacy
About
Jobs


Website

Models
Datasets
Spaces
Pricing
Docs

ChatGPT Writer
