# Supplementary material for: Automatic classification of mobile apps to ensure safe usage for adolescents
Source: PLoS One. 2025 Jan 16;20(1):e0313953. doi: 10.1371/journal.pone.0313953 (PMC11737711; doi:10.1371/journal.pone.0313953)
Supplement: S1 Dataset — (ZIP) [file pone.0313953.s001.zip › sealuzh_app_reviews · Datasets at Hugging Face - WebPage_files/inner.html]

StripeM-Inner 
