# Supplementary figures and images for: Automatic classification of mobile apps to ensure safe usage for adolescents
Source: PLoS One. 2025 Jan 16;20(1):e0313953. doi: 10.1371/journal.pone.0313953 (PMC11737711; doi:10.1371/journal.pone.0313953)

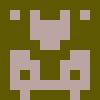

Supplement: S1 Dataset — (ZIP) [file pone.0313953.s001.zip › sealuzh_app_reviews · Datasets at Hugging Face - WebPage_files/119fa5e3bd9cccc0c434fed0017d6e43]

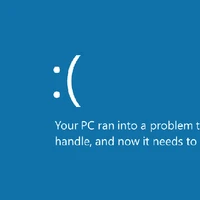

Supplement: S1 Dataset — (ZIP) [file pone.0313953.s001.zip › sealuzh_app_reviews · Datasets at Hugging Face - WebPage_files/HENl3spVJqmq4hHK9RGRq.png]

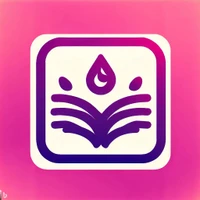

Supplement: S1 Dataset — (ZIP) [file pone.0313953.s001.zip › sealuzh_app_reviews · Datasets at Hugging Face - WebPage_files/j06-U5e2Tifi2xOnTudqS.jpeg]
